# Amputation of a Bull’s Penis1Presented at the Fourteenth Annual Meeting of the Iowa State Veterinary Medical Association, Des Moines, Iowa, February 11 and 12, 1902.

**Published:** 1902-05

**Authors:** George M. Walrod

**Affiliations:** Storm Lake, Iowa


### AMPUTATION OF A BULL'S PENIS.[1]

By George M. Walrod, V.S.,
STORM LAKE, IOWA.

On September 25, 1901, I was called to see a bull which the owner said had hurt his penis. On arriving I found the bull with his penis protruding about five or six inches, and split at the end for a distance of about one and one-half inches. On further examination I found the penis very much enlarged for about five or six inches inside the sheath, and gangrenous. My prognosis was that I might save the animal, but not the penis. The owner told me to do as I thought best, so I concluded to amputate the penis. I divided the sheath from the prepuce back to near the scrotum, and then divided the tissues of the penis until I came to the urethra, ligating the external and internal pudic arteries. I then dissected out one-half inch of the urethra and cut through it at right angles to its long axis, so as to leave the stub of the urethra about one-half inch longer than the remnant of the penis; then I divided the protruding part of the urethra into three equal parts, suturing each part to the corpus spongiosum. I then dissected the mucous lining from the sheath and excised a little of the sheath

[1] Presented at the Fourteenth Annual Meeting of the Iowa State Veterinary Medical Association, Des Moines, Iowa, February 11 and 12, 1902.

at the prepuce. I then sutured the skin incision which had been made in the sheath. Antiseptic precautions were, of course, taken throughout. An antiseptic wash was used for a few days after the operation, and this was followed by an ointment composed of iodoform and petrolatum, 1 to 8, applied to the rudimentary penis. The animal made a good recovery, without missing a feed. He was stall-fed for three months, at the end of which time he was quite fat, and was sold for beef.

### Discussion.

Dr. George A. Scott moved that the discussions be limited to twenty minutes. Seconded, put to vote, and carried.

Dr. Heck asked if the members thought that amputation of the horse's penis could be done with the animal in the standing position. He said he was thinking of trying it, by the aid of cocaine locally, with the horse standing. None of the members had had experience with the horse standing, but those who expressed themselves thought it could not be done in that way, and that the animal should be cast.

